# Detection and Location of Multi-Period Phenomena in Chaotic Binary Sequences

**DOI:** 10.3390/e24030331

**Published:** 2022-02-25

**Authors:** Chunlei Fan, Qun Ding

**Affiliations:** Electrical Engineering College, Heilongjiang University, Harbin 150080, China; 1984008@hlju.edu.cn

**Keywords:** randomness test, local periodicity, chaotic binary sequence, pseudo-random bit generator

## Abstract

Due to the influence of finite calculation accuracy and binary quantization method, the performance of chaotic binary sequences has been degraded in varying degrees, and some sequences emerge as multi-period phenomena. Aiming at the problem that it is difficult to accurately detect this phenomenon, this paper proposes a multi-period positioning algorithm (MPPA), which can accurately detect and locate the accurate period and local period phenomena contained in chaotic binary sequences. In order to test the effectiveness and correctness of the algorithm, the multi-period characteristics of logistic binary sequences with different calculation accuracy are analyzed. MPPA evaluates the randomness of binary sequences from a new perspective, which provides a new idea for the analysis of cryptographic security of chaotic sequences.

## 1. Introduction

At present, with the rapid development of embedded technology, mobile communications, and the Internet, information security has become increasingly significant. As one of the important means to ensure information security, the encryption algorithm has been widely used. Among the symmetric encryption algorithms, serial ciphers maintain advantages in wireless secure communications and dedicated encryption machines due to their simple structure, easy hardware implementation, limited error propagation, and fast encryption speed. Sequence ciphers are also called stream ciphers. For such ciphers, the pseudo-random bit generator (PRBG) is its core component. The quality of a stream cipher mainly depends on the complexity and randomness of the binary sequence (i.e., keystream) generated by PRBG. Traditional PRBGs are generally constructed based on m-sequence or M-sequence, which has good noise-like, autocorrelation, run-length distribution, and 0–1 balance. However, m-sequences are constructed based on linear feedback shift registers and primitive polynomials, and their linearity is easy to be cracked. The construction of nonlinear M-sequences has not yet been completely solved theoretically, and there are few construction methods. On the other hand, while traditional cryptography is being studied, chaotic systems have attracted extensive attention from relevant scholars because of their good characteristics such as initial value sensitivity, noise-like, topological transitivity, ergodicity, and long-term unpredictability [1]. Using chaos theory to design PRBG has become a new direction of current research. In recent years, related scholars have also proposed a variety of PRBGs based on chaotic systems [2,3,4]. The pros and cons of the chaotic pseudo-random sequence generator mainly depend on the randomness and complexity of the chaotic binary sequence. Therefore, a comprehensive evaluation of the performance of chaotic binary sequences has become a crucial criterion for measuring the security of chaotic ciphers.

In fact, as early as 1993, Stamp [5] studied the complexity measurement of 0–1 binary sequences and proposed a *k*-error linear complexity to analyze the sequence performance. In ref. [6], Feng et al. also innovatively designed a fixed period distance to measure the stability of the binary sequence. Subsequently, Elfeky et al. [7] designed a period mining algorithm to monitor the period phenomenon of discrete time series in real time. In 2005, when studying the linear complexity and stability of periodic sequences, Niu et al. [8] proposed a basic framework based on lattice structure to analyze the performance of sequences. In 2012, Ahadpour et al. [9] proposed the topological properties of the binary visibility graph as a randomness criterion from the complex network’s perspective. In 2019, Fatih et al. [10] put forward a new family of statistical randomness tests for a collection of binary sequences. This test can analyze periodic templates of binary sequences and evaluate the performance of the sequence. At present, related scholars generally use common detection methods for performance analysis of chaotic binary sequences, such as autocorrelation detection, discrete Fourier transform, linear complexity [11,12], entropy theory [13,14,15], frequency test, etc. In addition, several statistical test suites for binary sequences have also been developed successively, such as TestU01 [16], NIST SP800-22 [17], Diehard [18], and GM/T 0005-2012. These test specifications contain several detection items, which can analyze and evaluate the randomness of binary sequences from different angles.

However, due to the limited calculation accuracy of hardware equipment, binary quantization methods and rounding errors, the performance of chaotic binary sequences has been degraded to varying degrees [19,20,21,22]. In recent years, scholars in related fields have surprisingly discovered that some chaotic binary sequences emerge as locally weak random phenomena, that is, a measurable short sequence will periodically appear in a certain local range of the chaotic binary sequence. The local instability of the chaotic binary sequence is easily ignored. From the perspective of encryption, the appearance of this phenomenon is bound to affect the security and anti-attack ability of chaotic sequence ciphers. This phenomenon is difficult to locate and evaluate accurately by employing commonly used detection methods such as frequency detection, autocorrelation, poker detection, approximate entropy, overlapping subsequence, and binary derivation. Based on the above problems, Zheng et al. [23] proposed a binary sequence cycle monitoring method based on run-length characteristics to locate multiple periodic phenomena in a chaotic binary sequence. Nevertheless, this method can only detect part of the periodic phenomenon contained in the chaotic binary sequence, and the weak random phenomenon with a short period template cannot be accurately located. In order to compensate for the shortcomings of the aforementioned method, we designed a multi-period positioning algorithm (MPPA) based on the periodic detection matrix and monotonic stack technology. This algorithm can precisely locate the accurate period and local period phenomena contained in the chaotic binary sequence. Related experimental results also demonstrate the effectiveness and correctness of the proposed algorithm. MPPA enriches the set of randomness detection methods for chaotic binary sequences and provides a new idea for the analysis of the security of chaotic stream ciphers.

## 2. Local Periodicity and Mathematical Definition of Chaotic Binary Sequences

### 2.1. Local Periodicity

**Figure 1:**
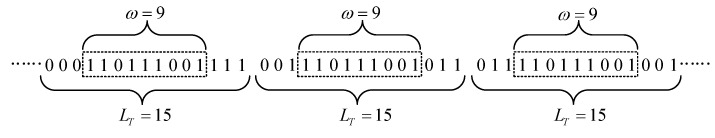
Local periodic phenomena of chaotic binary sequence.

As is well known, the classical chaos theory is defined in the continuous domain. In this case, there is no doubt that the chaotic sequence is aperiodic. However, when the chaotic system is implemented on a microprocessor with limited calculation accuracies such as in FPGA or DSP, the digitized chaotic system will be limited to a finite domain. According to the pigeon nest principle, it is not difficult to find that the original aperiodic chaotic sequence will undergo dynamic degradation and make it periodic. Assuming that ε(t) is a chaotic binary sequence, if ε(t) satisfies the formula $\varepsilon (t)=\varepsilon (t+kL_{T})$, the binary sequence has a traditional periodic phenomenon with length $L_{T}$. However, in some cases, a weak randomness problem will appear in the local range of the chaotic binary sequence (Figure 1), which is called the local periodicity of the binary sequence in this paper. According to the figure, a certain part of the quantized chaotic binary sequence is truncated, and the same bit string (110111001) of *ω* bits can appear periodically in each $L_{T}$ bit. This bit string is called the periodic template with a length of *ω* bits. 

It is difficult to accurately locate and analyze the locally weak random phenomenon of the chaotic binary sequence using conventional test methods such as discrete Fourier transform, poker detection, overlapping subsequence detection, approximate entropy, etc. The emergence of this locally weak random phenomenon is bound to have a negative impact on the randomness of chaotic binary sequences. Using such a binary sequence as the keystream of a sequence cipher will reduce the anti-decipherability of the ciphertext. Therefore, an effective multi-period detection algorithm plays a significant role in promoting the practical engineering application of chaos theory.

### 2.2. Mathematical Definition

In this section, according to the logical relationship contained in a chaotic binary sequence, a strict mathematical definition of the local periodicity in a binary sequence is proposed. This definition includes the period in the traditional sense, in addition to the above local periodic phenomenon. Furthermore, suppose there are consecutive positive integer sequences $\left\{\alpha_{1},\cdots ,\alpha_{i},\cdots ,\alpha_{\omega }\right\}$ and $\left\{\beta_{1},\cdots ,\beta_{j},\cdots ,\beta_{v}\right\}$ satisfying $\left\{\alpha_{i}|0<\alpha_{1}\;\leq \;\alpha_{i}\;\leq \;\alpha_{\omega }\;\leq \;L_{T},\;L_{T}\in {\mathbb{N}}^{*},\omega \in \left[1,L_{T}\right],1\;\leq \;i\;\leq \;\omega \right\}$ and $\left\{\beta_{j}|0<\beta_{1}\;\leq \;\beta_{j}\;\leq \beta_{v}<\left(L_{n}-L_{1}+1\right)/L_{T},L_{T}\in {\mathbb{N}}^{*},1\;\leq j\;\leq v\right\},$ respectively. Then, for arbitrary $\alpha_{i}$ and $\beta_{j}$ satisfying

(1) $$\varepsilon (L_{1}-1+\alpha_{i}+(\beta_{j}-1)\cdot L_{T})=\varepsilon (L_{1}-1+\alpha_{i}+\beta_{j}\cdot L_{T})$$

We can state that the binary sequence *ε*(*t*) exhibits a generalized periodicity with period length $L_{T}$ in the interval [$L_{1},L_{n}$], where *t* represents the relative position in the binary sequence *ε*(*t*).

(1) If $\omega =L_{T}$ and $v=\lceil \left(L_{n}-L_{1}+1\right)/L_{T}\rceil -1$, where ⌈·⌉ denotes a numerical rounding operation, Equation (1) describes a traditional periodicity with the period length $L_{T}$.
(2) If $0<\omega <L_{T}$ and $v\to \lceil \left(L_{n}-L_{1}+1\right)/L_{T}\rceil -1$, Equation (1) describes a local periodicity. Furthermore, the period template of the binary sequence *ε*(*t*) can be expressed as $\varepsilon \left(L_{1}-1+\alpha_{1}+\beta_{j}\cdot L_{T}\right)\varepsilon \left(L_{1}-1+\alpha_{2}+\beta_{j}\cdot L_{T}\right)\cdots \varepsilon \left(L_{1}-1+\alpha_{\omega }+\beta_{j}\cdot L_{T}\right)$, and its length is ω.

## 3. A Multi-Period Positioning Algorithm

In order to effectively detect the existence of the local periodic phenomenon in a binary sequence, that is to say, whether a weak randomness problem occurs in the local range of this sequence, in this section, we propose a multi-period positioning algorithm (MPPA) to enrich the set of randomness detection methods for binary sequences. The specific implementation steps of this algorithm are as follows:

**Step 1.** First, for an arbitrary chaotic binary sequence ε(t) with length n, we define a (n−1)×(n−1) two-dimensional matrix Q as the sequence reconstruction matrix of the binary sequence ε(t). $Q_{i,j}$ is the corresponding element in matrix Q and satisfies the equation $Q_{i,j}=\varepsilon (i+1)⊙\varepsilon (j)$. Furthermore, the sequence reconstruction matrix Q is defined as

(2) $$Q=\left[\begin{array}{ccccc} Q_{1,1} & & & & \\ Q_{2,1} & Q_{2,2} & & & \\ \vdots & \vdots & \ddots & & \\ Q_{n-2,1} & Q_{n-2,2} & \cdots & Q_{n-2,n-2} & \\ Q_{n-1,1} & Q_{n-1,2} & \cdots & Q_{n-1,n-2} & Q_{n-1,n-1} \end{array}\right]$$

where the symbol ⊙ denotes inclusive or logic operation. ε(i+1) and ε(j) are the ${\left(i+1\right)}^{\mathrm{th}}$ and $j^{\mathrm{th}}$ elements of the chaotic binary sequence ε(t) respectively. Here, we define the main diagonal of matrix Q as the first diagonal, which is composed of an element set $\left\{Q_{1,1},Q_{2,2},\cdots ,Q_{n-1,n-1}\right\}$. The diagonal formed by the element set $\left\{Q_{2,1},Q_{3,2},\cdots ,Q_{n-1,n-2}\right\}$ is the second diagonal of matrix Q. By analogy, matrix Q can form n−1 diagonal lines. Moreover, if the binary sequence ε(t) satisfies the equation ε(i)ε(i+1)⋯ε(i+ω−1)=ε(i+j)ε(i+j+1)⋯ε(i+j+ω−1), there is a run of ones with the length ω between the $i^{\mathrm{th}}$ and ${\left(i+\omega -1\right)}^{\mathrm{th}}$ elements of the $j^{\mathrm{th}}$ diagonal line of matrix Q. Obviously, matrix Q can reflect the local periodic phenomenon of sequence ε(t) to some extent.

**Step 2** Next, we can assume that $\left\{S_{k}^{i}\right\}$ is equivalent to the $k^{\mathrm{th}}$ diagonal line of matrix Q, where $1\leq i\leq L_{k}$ and $S_{k}^{i}\in \left\{0,1\right\}$. $L_{k}$ is the number of elements contained in $\left\{S_{k}^{i}\right\}$, i.e., the length of the $k^{\mathrm{th}}$ diagonal line. Furthermore, a r×k period detection matrix R can be generated by folding $\left\{S_{k}^{i}\right\}$ with period k. Here, if the length n of sequence ε(t) is even, k∈{2,3,⋯,n/2}. If n is odd, k∈{2,3,⋯,(n−1)/2}. Then, the variable r can be given as

(3) $$r=\left\{\begin{array}{cc} L_{k}/k, & L_{k}\mathrm{mod}k=0\\ \left\lfloor L_{k}/k\right\rfloor , & L_{k}\mathrm{mod}k>0 \end{array}\right.$$

where $\left\lfloor \cdot \right\rfloor$ denotes rounding down operation. Based on the above process, the matrix R can be constructed to detect the existence of traditional and local periodic phenomena in a binary sequence ε(t). The two-dimensional matrix R can be expressed as

(4) $$R=\left[\begin{array}{cccc} R_{1,1} & R_{1,2} & \cdots & R_{1,k}\\ R_{2,1} & R_{2,2} & \cdots & R_{2,k}\\ \vdots & \vdots & & \vdots \\ R_{r,1} & R_{r,2} & \cdots & R_{r,k} \end{array}\right]=\left[\begin{array}{cccc} S_{k}^{1} & S_{k}^{2} & \cdots & S_{k}^{k}\\ S_{k}^{k+1} & S_{k}^{k+2} & \cdots & S_{k}^{2k}\\ \vdots & \vdots & & \vdots \\ S_{k}^{(r-1)\cdot k+1} & S_{k}^{(r-1)\cdot k+2} & \cdots & S_{k}^{r\cdot k} \end{array}\right]$$

Then, the matrix R is further operated to obtain the final period detection matrix Y, and the mathematical expression of matrix Y can be defined as

(5) $$Y=\left[\begin{array}{cccc} Y_{1,1} & Y_{1,2} & \cdots & Y_{1,k}\\ Y_{2,1} & Y_{2,2} & \cdots & Y_{2,k}\\ \vdots & \vdots & & \vdots \\ Y_{r,1} & Y_{r,2} & \cdots & Y_{r,k} \end{array}\right]$$

The following correlation exists between the matrices R and Y:

(1) Assume that $R_{i,j}$ (1≤i≤r,1≤j≤k) is any element in matrix R. If i=1, $Y_{1,j}=R_{1,j}$;
(2) For element $R_{i,j}$ (i>1), if $R_{i,j}=0$, $Y_{i,j}=0$. In contrast, if $R_{i,j}=1$, we observe the number of consecutive 1s above the element $R_{i,j}$ in the $j^{\mathrm{th}}$ column of matrix R. If the number of consecutive 1s is γ, then $Y_{i,j}=\gamma +1$. Here, we can exemplify the above relationship, suppose the two-dimensional matrix R is
  (6) $$R=\left[\begin{array}{lllll} 0 & 1 & 1 & 0 & 0\\ 0 & 1 & 1 & 1 & 1\\ 0 & 0 & 1 & 1 & 0\\ 0 & 0 & 1 & 0 & 1 \end{array}\right]$$
  Next, the period detection matrix Y can be obtained as follows:
  (7) $$Y=\left[\begin{array}{lllll} 0 & 1 & 1 & 0 & 0\\ 0 & 2 & 2 & 1 & 1\\ 0 & 0 & 3 & 2 & 0\\ 0 & 0 & 4 & 0 & 1 \end{array}\right]$$

**Step 3.** Based on the period detection matrix Y, the multi-period phenomena contained in the chaotic binary sequence ε(t) can be detected and located. First, the last row element in matrix Y is extracted to construct a one-dimensional vector $V=\left[\begin{array}{llll} Y_{r,1} & Y_{r,2} & \cdots & Y_{r,k} \end{array}\right]$. After that, the value of the elements in the vector V is successively judged. If each element of vector V is equal to r, then the binary sequence ε(t) has an exact periodic phenomenon with length k. If an exact period is detected in the binary sequence ε(t), we do not need to detect the local period phenomenon. If the binary sequence does not contain the exact period, it is necessary to further check whether there is a local periodicity in the local range of the sequence ε(t). 

Next, the monotone stack theory can be used to detect and locate the local periodicity of the binary sequence ε(t). A monotone stack is a data structure, which is divided into monotone increasing stack and monotone decreasing stack. The specific execution process of local periodic detection is to scan the elements in the periodic detection matrix Y row by row to search for continuous nonzero element segments. Suppose that there is a nonzero element segment $U_{1}=\left[\begin{array}{llll} Y_{i,p} & Y_{i,p+1} & \cdots & Y_{i,p+l-1} \end{array}\right]$ with length l in the $i^{\mathrm{th}}$ row, that is, each element in the element segment $U_{1}$ is a positive integer. Furthermore, a zero element is added to the tail of the element segment $U_{1}$ to construct a new element segment $U_{2}=\left[\begin{array}{lllll} u(0) & u(1) & \cdots & u(l-1) & u(l) \end{array}\right]$, with length l+1, where u(l)=0. After performing the above operations, $U_{2}$ can be used as the input parameter of the local periodic positioning algorithm (LPPA) to judge whether there is a local periodicity in this range. The LPPA is presented as Algorithm 1 in pseudocode. In this algorithm, variable *thres* denotes the decision threshold of local period and thres∈(0,1). The larger the decision threshold is, the more obvious the local periodicity of the chaotic binary sequence is. In general, the decision threshold should be greater than or equal to 0.03. 

LPPA adopts monotonic decreasing stack technology. A set of key values (*c*-*Width*, *c*-1, *High*) can be obtained after each execution of the local cycle positioning algorithm. Based on this set of key values and the column coordinate *p* of the first element of $U_{1}$, we can deduce the start point (*Start* = *p* + *c*-*Width*) and the endpoint (*End* = *p* + *c*-1) of the period template. Furthermore, the precise location of the local period contained in the chaotic binary sequence ε(t) can be expressed as

(8) $$\begin{array}{cc} \begin{array}{l} \varepsilon ((i-High)\cdot k+Start)\cdots \varepsilon ((i-High)\cdot k+End)=\\ \varepsilon ((i-High+h)\cdot k+Start)\cdots \varepsilon ((i-High+h)\cdot k+End), \end{array} & h\in \{1,2,\cdots ,High\} \end{array}$$

Based on the above operation steps, we can accurately locate whether there is an exact period and local period in any chaotic binary sequence. This algorithm evaluates the randomness of chaotic binary sequences from a new perspective and then compensates for the shortcomings of existing randomness detection methods.
**Algorithm 1** Local periodic positioning algorithm (LPPA)1: Define a stack, the width, height, and area of the “1” block: Stack, *Width*, *High*, *BlockArea*
2: **for**
*c* from 0 to *l*
**do**
3:    **while** !Stack.isEmpty() && *u*(stack.top()) > *u*(*c*) **do**
4:      *High*←*u*(stack.top());//stack.top() denotes stack top. 5:      **while** !Stack.isEmpty() && *High* == *u*(stack.top()) **do**
6:        Stack.pop(); 7:      **end while**
8:      *Width*←0; 9:      **if** Stack.isEmpty() **then**
10:          *Width*←*c*; 11:       **else**
12:          *Width*←*c*-Stack.top()-1; 13:      **end if**
14:      *BlockArea*←Width*High; 15:      **if** (*BlockArea* + *Width*) >= n*thres
**then**
16:         Output a set of variables (*c*-*Width*, *c*-1, *High*); 17:      **end if**
18:    **end while**
19:    Stack.push(*c*); 20: **end for**

## 4. Randomness Tests of Chaotic Binary Sequences

In this section, in order to verify the effectiveness and practicability of the proposed algorithm, MPPA and several commonly used detection methods are performed to comprehensively test the performance of logistic chaotic binary sequences under different calculation accuracy. The iterative equation of the digital logistic map in integer domain [24] is given by

(9) $$\begin{array}{cc} z_{n+1}=\left\lfloor \mu z_{n}(1-\frac{z_{n}}{2^{m}})\right\rfloor , & z_{n}\in [0,2^{m}-1] \end{array}$$

where $\left\lfloor \cdot \right\rfloor$ denotes rounding down operation; μ and m represent the control parameter and the finite computational precision, respectively. We adopted incremental quantization to binarize the discrete logistic sequence. Incremental quantization is also called direction quantization, which is defined as

(10) $$s_{n}=\left\{\begin{array}{cc} 0 & \Delta z<0\\ 1 & \Delta z\geq 0 \end{array}\right.$$

where $\Delta z=z_{n+1}-z_{n}$, that is, the difference between two adjacent chaotic iteration values. If Δz is less than 0, the result of quantization is 0; otherwise, the result is 1. $\left\{z_{n}\right\}$ and $\left\{s_{n}\right\}$ are the chaotic real-valued sequence and the quantized binary sequence, respectively. Obviously, the chaotic binary sequence generated by the incremental quantization method reflects the motion trajectory of the chaotic map to a certain extent. Based on Equation (10), we used incremental quantization to generate different logistic binary sequences, with calculation accuracies of *m* = 8, 16, and 32. In addition, numerical simulations were performed to assess the performance of the logistic binary sequences in terms of autocorrelation, permutation entropy, linear complexity, and MPPA.

### 4.1. Autocorrelation Test

**Figure 2:**
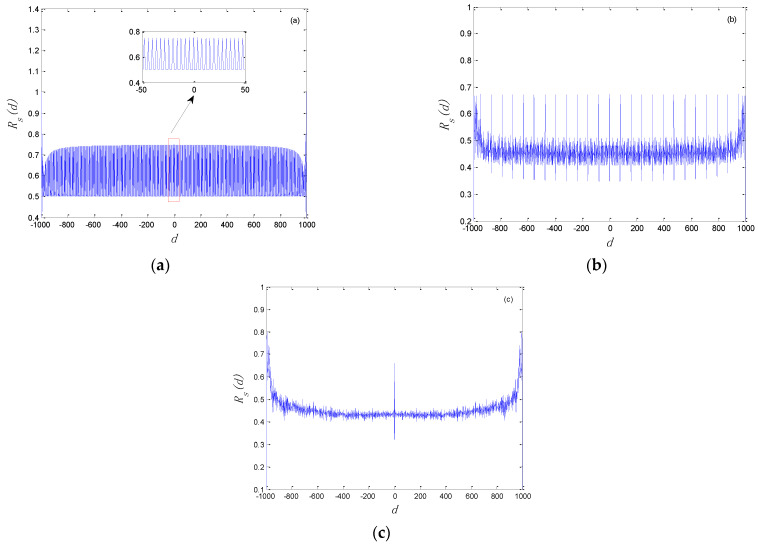
Autocorrelation test of logistic binary sequences: (**a**) m=8; (**b**) m=16; (**c**) m=32.

The autocorrelation test was used to detect the correlation degree between the binary sequence $\left\{s_{n}\right\}$ and the new sequence obtained by shifting the sequence to the left by *d* bits. The test method is generally realized by the autocorrelation function of the binary sequence $\left\{s_{n}\right\}$, and its mathematical formula can be expressed as

(11) $$R_{s}(d)=\frac{1}{N-\left|d\right|}\sum_{n=0}^{N-1-\left|d\right|}s_{n}s_{n+d}$$

where $R_{s}(d)$ and N denote autocorrelation function and the length of the chaotic binary sequence, respectively. Based on Equation (11), an autocorrelation test was carried out for logistic binary sequences with different calculation accuracy, and simulation results are shown in Figure 2. According to Figure 2a, when the calculation accuracy *m* is equal to 8, the autocorrelation function of the logistic binary sequence has extremely dense peaks with approximately equal amplitude, which indicates that the binary sequence has strong autocorrelation and short periodicity. When the calculation accuracy *m* = 16, the peak density of the autocorrelation function for the binary sequence with a length of 1000 is reduced. Here, it still has multiple spectral lines, which still shows a certain degree of correlation and periodicity. From Figure 2c, we can observe that the waveform of the autocorrelation function is like an impulse function δ(t). From the perspective of the autocorrelation test, the chaotic binary sequence has good randomness. In fact, there are still local weak random features.

### 4.2. Permutation Entropy

In 2002, Bandt and Pompe [25] proposed a measurement method to analyze the complexity of discrete time series—namely, permutation entropy (PE). This method has many advantages, such as strong robustness, fast-computing speed, and invariance of nonlinear monotonic transformation. Therefore, it has been widely used to evaluate sequence complexity in recent years. The range of permutation entropy is between 0 and 1. The larger the value, the better the randomness, and the higher the complexity of the discrete chaotic sequence. In this section, the permutation entropy of the logistic binary sequences with different calculation accuracy is calculated. The two parameters of PE (i.e., embedding dimension θ and delay time τ) are set as 6 and 1, respectively. The experimental results are listed in Table 1. According to Table 1, with the increase in the calculation precision, the permutation entropy of the logistic binary sequences also gradually increases. When the calculation precision *m* is 16 or 32, the permutation entropy is better than the PE value of the logistic binary sequence with the precision *m* = 8. However, when the calculation precision *m* = 32, the permutation entropy of the binary sequence is only 0.44276, which is still far from the ideal value of 1.

### 4.3. Linear Complexity

**Figure 3:**
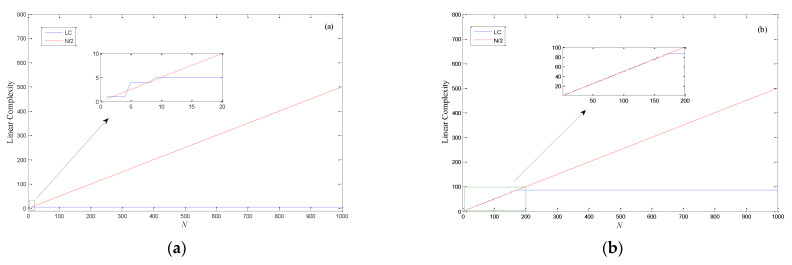

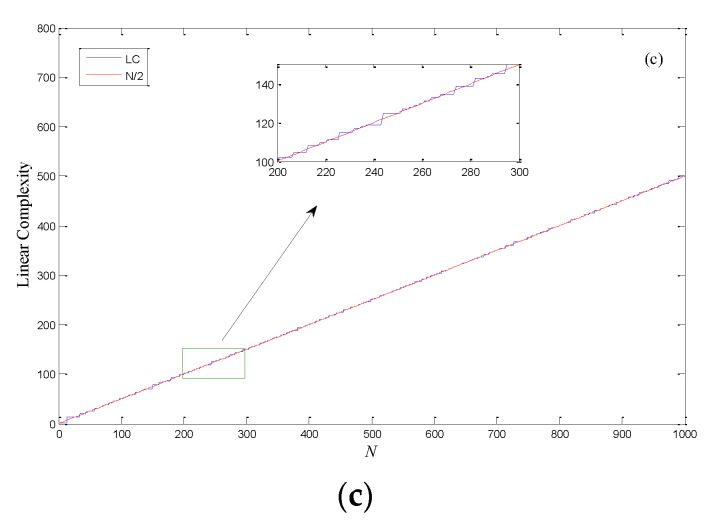
Linear complexity test of logistic binary sequences: (**a**) m=8; (**b**) m=16; (**c**) m=32.

Linear complexity (LC) is one of the important means to evaluate the performance of binary sequences. The linear complexity of binary sequences is usually calculated using the Berlekamp–Massey algorithm. The algorithm is very efficient for determining the linear complexity of a finite binary sequence. For a chaotic binary sequence with length *N*, if the linear complexity of the sequence is half of its length, it is considered that the binary sequence has desirable linear complexity. In this section, the linear complexity of logistic binary sequences with different calculation accuracy is calculated, and the numerical results are illustrated in Figure 3. As can be seen from Figure 3a, when the length of the logistic binary sequence exceeds 9 bits, its linear complexity is always equal to 5. That is to say, the binary sequence can be equivalently generated by constructing a five-order LFSR. Similarly, when the calculation accuracy *m* = 16, it can be equivalently generated by only constructing an 87-order LFSR. According to Figure 3b, when the length of the binary sequence increases, the linear complexity of the logistic binary sequence remains at a low value. Nevertheless, from Figure 3c, the value of linear complexity fluctuates up and down along the straight line *N*/2, which shows that the binary sequence has ideal linear complexity.

### 4.4. MPPA Test

**Figure 4:**
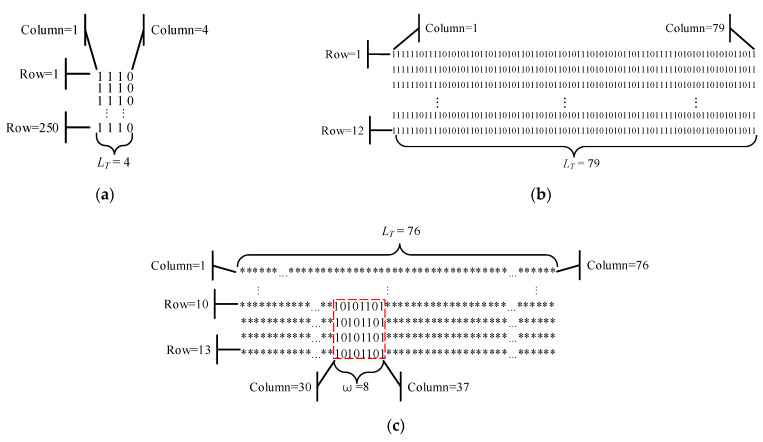
MPPA test of logistic binary sequences: (**a**) $f_{8}(11)$; (**b**) $f_{16}(43)$; (**c**) $f_{32}(4700)$.

The above-mentioned test methods analyze the performance of logistic binary sequences from different angles, but none of them can accurately locate and detect the locally weak randomness phenomenon of the binary sequence. In this section, the proposed algorithm (i.e., MPPA) is used to analyze the local randomness of chaotic binary sequences with length 1000, which can comprehensively evaluate the performance of binary sequences from a new perspective. For explanatory convenience, let us assume that $f_{m}(z_{0})$ denotes a logistic binary sequence, where *m* and $z_{0}$ represent the finite computational precision and the initial value of the digital logistic map. The decision threshold of MPPA is set as 0.03, and the experimental results are shown in Figure 4. It can be seen from Figure 4a,b that the logistic binary sequences $f_{8}(11)$ and $f_{16}(43)$ have exact periodic phenomena with period lengths $L_{T}=4$ and 79, respectively. According to Figure 4c, when the computational precision *m* = 32, we observe that the sequence $f_{32}(4700)$ contains a local period with length $L_{T}=76$. Moreover, the position of the period template ω (i.e., the bit string 10101101) can be precisely located between the 10th row, 30th column, and 13th row, 37th column. According to the results of the MPPA test, the logistic binary sequences have certain exact periodic and local periodic phenomena.

**Figure 5:**
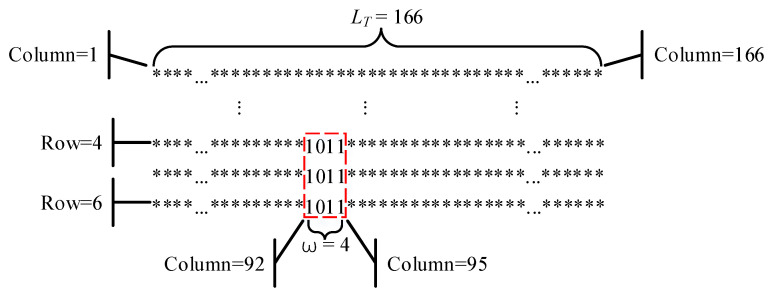
MPPA test of the improved logistic binary sequence with *thres* = 0.01.

The above experiment was designed to analyze the local randomness of the original logistic binary sequence under different calculation precisions. The experimental results show that it has obvious precise period and local period phenomenon. For comparison, we also carried out the MPPA test for the improved logistic sequence. As an example, the improved logistic sequence proposed in Ref. [22] can be used as a sequence to be detected. When the MPPA test was performed on the improved logistic binary sequence and the decision threshold *thres* was set to the recommended value (e.g., 0.03), the test result shows that the binary sequence has neither precise period nor local period phenomenon. Furthermore, the test result for when *thres* was set to a non-recommended value of 0.01 (small enough) is shown in Figure 5. It can be observed that the sequence contains an insignificant local period, with length $L_{T}=166$. The position of period template ω (the bit string 1011 is short enough) can be located between the 4th row, 92nd column, and 6th row, 95th column. The experimental results show that the binary sequence has no local weak random phenomenon under the recommended threshold value. Only when the *thres* is reduced to a non-recommended value of 0.01 can the sequence detect the local period with a small periodic template, which shows that the local periodic phenomenon of the improved binary sequence is not obvious and ignorable. In addition, we also conducted MPPA tests on some of the currently proposed robust chaotic generators (e.g., Refs. [26,27]), and the experimental results show that the correlated binary sequences do not exhibit obvious local periodicity. Based on the above experiments, we can conclude that the use of periodic cycle jumps, construction of high-dimensional chaotic systems with high Lyapunov exponent, and decorrelation transformation can effectively reduce the local periodic phenomenon of chaotic binary sequences and improve the randomness of sequences.

## 5. Discussion

To solve the problem of the difficult detection of locally weak randomness of chaotic binary sequences, a multi-period positioning algorithm is designed based on the periodic detection matrix and monotone stack technology. This algorithm is not limited by the length of the periodic template and can accurately detect and locate the accurate period and local period phenomena contained in chaotic binary sequences. Furthermore, the performance of logistic binary sequences with different calculation accuracy was analyzed in detail. From the experimental results, binary sequences with low calculation accuracy have obvious short periodicity, while binary sequences with high calculation accuracy have certain locally weak randomness, which cannot meet the requirements of sequence encryption. Compared with other detection methods such as autocorrelation, linear complexity, and permutation entropy, MPPA analyzes the performance of binary sequences from the perspective of local randomness, which compensates for the shortcomings of conventional detection methods. In future research, we should also seek an effective method to resist the local periodicity of the chaotic binary sequence, so as to pave the way for enhancing the security of the chaotic sequence cipher.

## Figures and Tables

**Table 1:** PEs of logistic binary sequences with various computational precisions.

Calculation Accuracy *m*	PE Value
8	0.21171
16	0.43414
32	0.44276

## Data Availability

Not applicable.
